# Supplementary material for: End Sequence Analysis Toolkit (ESAT) expands the extractable information from single-cell RNA-seq data
Source: Genome Res. 2016 Oct;26(10):1397–410. doi: 10.1101/gr.207902.116 (PMC5052061; doi:10.1101/gr.207902.116)
Supplement: Supplemental Material [file supp_gr.207902.116_Supp_Materials.tar.gz › ESAT-esat_v0.1/doc/html/classbroad_1_1core_1_1annotation_1_1_repeat_masker_annotation-members.html]

ESAT: Member List


ESAT


- Main Page
- Related Pages
- Packages
- Classes
- Files

- Class List
- Class Index
- Class Hierarchy
- Class Members

All Classes Namespaces Files Functions Variables Enumerator Pages

- broad
- core
- annotation
- RepeatMaskerAnnotation

broad.core.annotation.RepeatMaskerAnnotation Member List

This is the complete list of members for broad.core.annotation.RepeatMaskerAnnotation, including all inherited members.

|  |  |  |
| --- | --- | --- |
| addBlock(String name, int start, int end) | broad.core.annotation.BasicGenomicAnnotation |  |
| addExtraScore(double score) | broad.core.annotation.BasicLightweightAnnotation |  |
| almostEqual(LightweightGenomicAnnotation other, int fudge) | broad.core.annotation.BasicLightweightAnnotation |  |
| BasicGenomicAnnotation() | broad.core.annotation.BasicGenomicAnnotation | protected |
| BasicGenomicAnnotation(String name) | broad.core.annotation.BasicGenomicAnnotation |  |
| BasicGenomicAnnotation(String name, String chr, int start, int end) | broad.core.annotation.BasicGenomicAnnotation |  |
| BasicGenomicAnnotation(String name, String chr, int start, int end, Strand strand) | broad.core.annotation.BasicGenomicAnnotation |  |
| BasicGenomicAnnotation(LightweightGenomicAnnotation ga) | broad.core.annotation.BasicGenomicAnnotation |  |
| BasicGenomicAnnotation(Annotation ga) | broad.core.annotation.BasicGenomicAnnotation |  |
| BasicGenomicAnnotation(GenomicAnnotation ga) | broad.core.annotation.BasicGenomicAnnotation |  |
| BasicGenomicAnnotation(String[] info) | broad.core.annotation.BasicGenomicAnnotation |  |
| BasicLightweightAnnotation() | broad.core.annotation.BasicLightweightAnnotation |  |
| BasicLightweightAnnotation(String chr, int start, int end) | broad.core.annotation.BasicLightweightAnnotation |  |
| BasicLightweightAnnotation(String chr, int start, int end, String orientation) | broad.core.annotation.BasicLightweightAnnotation |  |
| BasicLightweightAnnotation(String chr, int start, int end, Strand orientation, String name) | broad.core.annotation.BasicLightweightAnnotation |  |
| BasicLightweightAnnotation(String chr, int start, int end, String orientation, double Scr) | broad.core.annotation.BasicLightweightAnnotation |  |
| BasicLightweightAnnotation(String chr, String start, String end) | broad.core.annotation.BasicLightweightAnnotation |  |
| BasicLightweightAnnotation(LightweightGenomicAnnotation annotation) | broad.core.annotation.BasicLightweightAnnotation |  |
| BasicLightweightAnnotation(Annotation annotation) | broad.core.annotation.BasicLightweightAnnotation |  |
| compareTo(GenomicAnnotation arg0) | broad.core.annotation.BasicGenomicAnnotation |  |
| broad::core::annotation::BasicLightweightAnnotation.compareTo(LightweightGenomicAnnotation b) | broad.core.annotation.BasicLightweightAnnotation |  |
| compareToAnnotation(Annotation b) | umms.core.annotation.Annotation |  |
| complement() | umms.core.annotation.Annotation |  |
| contains(LightweightGenomicAnnotation other) | broad.core.annotation.BasicLightweightAnnotation |  |
| umms::core::annotation::Annotation.contains(Annotation other) | umms.core.annotation.Annotation |  |
| copy() | umms.core.annotation.Annotation |  |
| createFromUCSC(String ucsc) | broad.core.annotation.BasicLightweightAnnotation | static |
| disect(Annotation a) | broad.core.annotation.BasicGenomicAnnotation |  |
| disect(List<?extends Annotation > disectors) | broad.core.annotation.BasicGenomicAnnotation |  |
| equals(Annotation other) | umms.core.annotation.Annotation |  |
| equals(Annotation other, boolean useOrientation) | umms.core.annotation.Annotation |  |
| expand(int deltaStart, int deltaEnd) | umms.core.annotation.Annotation |  |
| fullyContains(Annotation annotation) | umms.core.annotation.Annotation |  |
| broad::core::annotation::BasicLightweightAnnotation.getBlocks() | umms.core.annotation.Annotation |  |
| broad::core::annotation::BasicLightweightAnnotation.getBlocks(boolean oriented) | umms.core.annotation.Annotation |  |
| broad::core::annotation::GenomicAnnotation.getBlocks() | broad.core.annotation.GenomicAnnotation |  |
| getChr() | umms.core.annotation.Annotation |  |
| getChromosome() | broad.core.annotation.BasicLightweightAnnotation |  |
| getChromosomeString() | broad.core.annotation.BasicLightweightAnnotation |  |
| getDistanceTo(LightweightGenomicAnnotation other) | broad.core.annotation.BasicLightweightAnnotation |  |
| umms::core::annotation::Annotation.getDistanceTo(Annotation other) | umms.core.annotation.Annotation |  |
| getEnd() | broad.core.annotation.LightweightGenomicAnnotation |  |
| getExtraScore(int i) | broad.core.annotation.BasicLightweightAnnotation |  |
| getExtraScores() | broad.core.annotation.BasicLightweightAnnotation |  |
| getFivePrimeBases() | broad.core.annotation.BasicGenomicAnnotation |  |
| getFullInfoString() | umms.core.annotation.Annotation |  |
| getId() | broad.core.annotation.BasicGenomicAnnotation |  |
| getLength() | broad.core.annotation.BasicGenomicAnnotation |  |
| getLengthOnReference() | umms.core.annotation.Annotation |  |
| broad::core::annotation::BasicLightweightAnnotation.getLocationString() | broad.core.annotation.BasicLightweightAnnotation |  |
| broad::core::annotation::GenomicAnnotation.getLocationString() | broad.core.annotation.GenomicAnnotation |  |
| getMiddle() | broad.core.annotation.BasicLightweightAnnotation |  |
| getMidpoint() | umms.core.annotation.Annotation |  |
| broad::core::annotation::BasicLightweightAnnotation.getName() | broad.core.annotation.LightweightGenomicAnnotation |  |
| broad::core::annotation::GenomicAnnotation.broad::core::annotation::Feature.getName() | broad.core.annotation.Feature |  |
| broad::core::annotation::BasicLightweightAnnotation.getOrientation() | broad.core.annotation.LightweightGenomicAnnotation |  |
| broad::core::annotation::GenomicAnnotation.getOrientation() | broad.core.annotation.GenomicAnnotation |  |
| getOrientedEnd() | broad.core.annotation.BasicGenomicAnnotation |  |
| getOrientedStart() | broad.core.annotation.BasicGenomicAnnotation |  |
| getOverlap(LightweightGenomicAnnotation other) | broad.core.annotation.BasicLightweightAnnotation |  |
| umms::core::annotation::Annotation.getOverlap(Annotation other) | umms.core.annotation.Annotation |  |
| getPositionAtReferenceCoordinate(int referenceCoordinate) | umms.core.annotation.Annotation |  |
| getPositionAtReferenceCoordinate(int referenceCoordinate, boolean ignoreOrientation) | umms.core.annotation.Annotation |  |
| getReferenceCoordinateAtPosition(int positionInAnnotation) | umms.core.annotation.Annotation |  |
| getReferenceCoordinateAtPosition(int positionInAnnotation, boolean ignoreOrientation) | umms.core.annotation.Annotation |  |
| getReferenceName() | umms.core.annotation.Annotation |  |
| getRepeatClass() | broad.core.annotation.RepeatMaskerAnnotation |  |
| getRepeatFamily() | broad.core.annotation.RepeatMaskerAnnotation |  |
| getRepeatName() | broad.core.annotation.RepeatMaskerAnnotation |  |
| getSAMEnd() | umms.core.annotation.Annotation |  |
| getSAMStart() | umms.core.annotation.Annotation |  |
| broad::core::annotation::BasicLightweightAnnotation.getScore() | broad.core.annotation.BasicLightweightAnnotation |  |
| broad::core::annotation::GenomicAnnotation.broad::core::annotation::Feature.getScore() | broad.core.annotation.Feature |  |
| getSequence() | broad.core.annotation.BasicGenomicAnnotation |  |
| getSize() | umms.core.annotation.Annotation |  |
| getSpliceConnections() | umms.core.annotation.Annotation |  |
| getStart() | broad.core.annotation.LightweightGenomicAnnotation |  |
| getStrand() | umms.core.annotation.Annotation |  |
| getThreePrimeBases() | broad.core.annotation.BasicGenomicAnnotation |  |
| hashCode() | broad.core.annotation.BasicLightweightAnnotation |  |
| hasOrientation() | umms.core.annotation.Annotation |  |
| inReversedOrientation() | broad.core.annotation.BasicLightweightAnnotation |  |
| intersect(LightweightGenomicAnnotation other) | broad.core.annotation.BasicLightweightAnnotation |  |
| intersect(Collection<?extends Annotation > annotations) | broad.core.annotation.BasicLightweightAnnotation |  |
| broad::core::annotation::LightweightGenomicAnnotation.intersect(Annotation other) | umms.core.annotation.Annotation |  |
| broad::core::annotation::LightweightGenomicAnnotation.intersect(List<?extends Annotation > annotations) | umms.core.annotation.Annotation |  |
| isFlankedBy(TwoSubjectAnnotation twoSubjectAnnotation, int buffer) | broad.core.annotation.BasicGenomicAnnotation |  |
| isNegativeStrand() | umms.core.annotation.Annotation |  |
| isUnoriented() | umms.core.annotation.Annotation |  |
| lastIdxWhereListSearchBroke() | broad.core.annotation.BasicGenomicAnnotation |  |
| length() | broad.core.annotation.BasicLightweightAnnotation |  |
| mayHaveBlocks() | broad.core.annotation.BasicGenomicAnnotation |  |
| mergeAllOverlappers(TreeSet< BasicLightweightAnnotation > regions) | broad.core.annotation.BasicLightweightAnnotation | static |
| minus(Annotation other) | umms.core.annotation.Annotation |  |
| minus(Collection<?extends Annotation > others) | umms.core.annotation.Annotation |  |
| moveToCoordinate(int coordinateInReference) | umms.core.annotation.Annotation |  |
| numBlocks() | umms.core.annotation.Annotation |  |
| overlaps(Collection<?extends Annotation > others, int buffer) | broad.core.annotation.BasicLightweightAnnotation |  |
| overlaps(Collection<?extends Annotation > others) | broad.core.annotation.BasicLightweightAnnotation |  |
| broad::core::annotation::LightweightGenomicAnnotation.overlaps(Annotation other, int buffer) | umms.core.annotation.Annotation |  |
| broad::core::annotation::LightweightGenomicAnnotation.overlaps(Annotation other) | umms.core.annotation.Annotation |  |
| broad::core::annotation::LightweightGenomicAnnotation.overlaps(Annotation other, int buffer, boolean considerOrientation) | umms.core.annotation.Annotation |  |
| broad::core::annotation::LightweightGenomicAnnotation.overlaps(Annotation other, boolean considerOrientation) | umms.core.annotation.Annotation |  |
| overlapsStranded(Annotation other) | umms.core.annotation.Annotation |  |
| reduceToDifference(GenomicAnnotation other) | broad.core.annotation.BasicGenomicAnnotation |  |
| removeExtraScores() | broad.core.annotation.BasicLightweightAnnotation |  |
| RepeatMaskerAnnotation() | broad.core.annotation.RepeatMaskerAnnotation |  |
| RepeatMaskerAnnotation(String name) | broad.core.annotation.RepeatMaskerAnnotation |  |
| RepeatMaskerAnnotation(GenomicAnnotation ga) | broad.core.annotation.RepeatMaskerAnnotation |  |
| RepeatMaskerAnnotation(String[] info) | broad.core.annotation.RepeatMaskerAnnotation |  |
| RepeatMaskerAnnotation(String chr, int start, int end, String strand, String repName, String repClass, String repFamily) | broad.core.annotation.RepeatMaskerAnnotation |  |
| RepeatMaskerAnnotation(int bin, int alignmentScore, int mismatches, int deleted, int inserted, String chr, int start, int end, int basesLeftAfterMatch, String strand, String repName, String repClass, String repFamily, int repStart, int repEnd, int repLeft) | broad.core.annotation.RepeatMaskerAnnotation |  |
| setBoundariesFromAnnoations(List<?extends GenomicAnnotation > annotations) | broad.core.annotation.BasicLightweightAnnotation |  |
| setChromosome(String chr) | broad.core.annotation.BasicLightweightAnnotation |  |
| setEnd(int end) | broad.core.annotation.LightweightGenomicAnnotation |  |
| setFivePrimeBuffer(int bufferSize) | broad.core.annotation.BasicGenomicAnnotation |  |
| setId(String id) | broad.core.annotation.BasicGenomicAnnotation |  |
| setName(String name) | broad.core.annotation.LightweightGenomicAnnotation |  |
| setOrientation(boolean orientation) | broad.core.annotation.BasicGenomicAnnotation |  |
| broad::core::annotation::BasicLightweightAnnotation.setOrientation(String orientation) | broad.core.annotation.BasicLightweightAnnotation |  |
| broad::core::annotation::BasicLightweightAnnotation.umms::core::annotation::Annotation.setOrientation(char orientation) | umms.core.annotation.Annotation |  |
| broad::core::annotation::BasicLightweightAnnotation.umms::core::annotation::Annotation.setOrientation(Strand orientation) | umms.core.annotation.Annotation |  |
| broad::core::annotation::GenomicAnnotation.setOrientation(String orientationString) | broad.core.annotation.GenomicAnnotation |  |
| setOrientedEnd(int orientedEnd) | umms.core.annotation.Annotation |  |
| setOrientedStart(int orientedStart) | umms.core.annotation.Annotation |  |
| setReferenceName(String refName) | umms.core.annotation.Annotation |  |
| broad::core::annotation::BasicLightweightAnnotation.setReversedOrientation(boolean isInReversedOrientation) | broad.core.annotation.BasicLightweightAnnotation |  |
| broad::core::annotation::GenomicAnnotation.setReversedOrientation(boolean isInReversedOrientation) | broad.core.annotation.GenomicAnnotation |  |
| setScore(double score) | broad.core.annotation.BasicLightweightAnnotation |  |
| setSequence(Sequence sequence) | broad.core.annotation.BasicGenomicAnnotation |  |
| setStart(String data) | broad.core.annotation.BasicLightweightAnnotation | protected |
| broad::core::annotation::LightweightGenomicAnnotation.setStart(int start) | broad.core.annotation.LightweightGenomicAnnotation |  |
| setThreePrimeBuffer(int bufferSize) | broad.core.annotation.BasicGenomicAnnotation |  |
| shift(int delta) | umms.core.annotation.Annotation |  |
| size() | umms.core.annotation.Annotation |  |
| stitchList(Collection<?extends Annotation > sortedList, int maxDistanceToStitch) | broad.core.annotation.BasicLightweightAnnotation | static |
| stitchTo(LightweightGenomicAnnotation other) | broad.core.annotation.BasicLightweightAnnotation |  |
| umms::core::annotation::Annotation.stitchTo(Annotation next) | umms.core.annotation.Annotation |  |
| takeIntersection(LightweightGenomicAnnotation other) | broad.core.annotation.BasicLightweightAnnotation |  |
| takeUnion(LightweightGenomicAnnotation other) | broad.core.annotation.BasicLightweightAnnotation |  |
| toBED() | umms.core.annotation.Annotation |  |
| toBED(int r, int g, int b) | umms.core.annotation.Annotation |  |
| toBEDGraph() | umms.core.annotation.Annotation |  |
| toCigar() | umms.core.annotation.Annotation |  |
| toShortBED() | umms.core.annotation.Annotation |  |
| toString() | broad.core.annotation.RepeatMaskerAnnotation |  |
| toUCSC() | broad.core.annotation.BasicLightweightAnnotation |  |
| trim(int deltaStart, int deltaEnd) | umms.core.annotation.Annotation |  |
| union(Annotation other) | umms.core.annotation.Annotation |  |


---

Generated on Fri Aug 15 2014 15:10:51 for ESAT by  

 1.8.7
